# Heterologous expression and antitumor activity analysis of syringolin from *Pseudomonas syringae* pv. *syringae* B728a

**DOI:** 10.1186/s12934-018-0859-1

**Published:** 2018-02-26

**Authors:** Fan Huang, Jianli Tang, Lian He, Xuezhi Ding, Shaoya Huang, Youming Zhang, Yunjun Sun, Liqiu Xia

**Affiliations:** Hunan Provincial Key Laboratory of Microbial Molecular Biology, State Key Laboratory of Developmental Biology of Freshwater Fish, College of Life Science, Hunan Normal University, Changsha, 410081 People’s Republic of China

**Keywords:** Antitumor, Heterologous expression, Red/ET recombineering, Syringolin, *Streptomyces*

## Abstract

**Background:**

Syringolin, synthesized by a mixed non-ribosomal peptide synthetase/polyketide synthetase in *Pseudomonas syringae* pv. *syringae* (*Pss*) B728a, is a novel eukaryotic proteasome inhibitor. Meanwhile, directly modifying large fragments in the PKS/NRPS gene cluster through traditional DNA engineering techniques is very difficult. In this study, we directly cloned the *syl* gene cluster from *Pss* B301D-R via Red/ET recombineering to effectively express syringolin in heterologous hosts.

**Results:**

A 22 kb genomic fragment containing the *sylA*–*sylE* gene cluster was cloned into the pASK vector, and the obtained recombinant plasmid was transferred into *Streptomyces coelicolor* and *Streptomyces lividans* for the heterologous expression of syringolin. Transcriptional levels of recombinant *syl* gene in *S. coelicolor* M145 and *S. lividans* TK24 were evaluated via RT-PCR and the production of syringolin compounds was detected via LC–MS analysis. The extracts of the engineered bacteria showed cytotoxic activity to B16, 4T1, Meth-A, and HeLa tumor cells. It is noteworthy that the syringolin displayed anticancer activity against C57BL/6 mice with B16 murine melanoma tumor cells. Together, our results herein demonstrate the potential of syrinolin as effective antitumor agent that can treat various cancers without apparent adverse effects.

**Conclusions:**

This present study is the first to report the heterologous expression of the entire *syl* gene cluster in *Streptomyces* strains and the successful expression of syringolin in both *S. coelicolor* M145 and *S. lividans* TK24. Syringolin derivatives demonstrated high cytotoxicity in vitro and in vivo. Hence, this paper provided an important foundation for the discovery and production of new antitumor compounds.

## Background

**Fig. 1:**
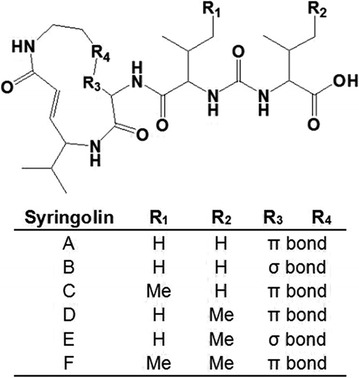
The structure of syringolin. The ring structure consists of two non-proteinogenic amino acids, namely, 3,4-dehydrolysine and 5-methyl-4-amino-2-hexenoic acid. The α-amino group of the latter attaches to valine through a peptide bond, which in turn, is linked to a second valine residue via an unusual ureido group

*Pseudomonas syringae* pv. *syringae* (*Pss*) is a foliar bacterial pathogen that causes brown spot disease in snap beans (*Phaseolus vulgaris L.*) [1, 2] and produces a novel polyketide complex, known as syringolin [3, 4]. Application of syringolin at micromolar concentrations onto rice plants can induce resistance against rice blast fungus. However, *Pss* mutants are incapable of syringolin biosynthesis and induce defense reactions and resistance, revealing its marginal therapeutic activity against systemic fungal infections [5, 6]. Recently, syringolin has shown potent in vivo antitumor activity against neuroblastoma, ovarian, and leukemic cancer cells [7]. Such specificity of syringolin inhibits all three catalytic activities of eukaryotic proteasomes [8, 9]. The proteasome acting during protein degradation has been known as a biological target for the clinic treatment lately [10, 11]. Syringolin was confirmed as the notable peptide moiety in a 12-membered macrolactam ring structure, which also has two double bonds with (E)-configuration and an unusual urea moiety [12] (Fig. 1). The special chain reversal by ureido linkage was also present in natural products, anabaenopeptins [13], brunsvicamides [14], pacidomycins [15], mureidomycins [16], and napsamycins [17]. Syringolin is a bioactive member of the syrbactins family, which also includes glidobactins and cepafungins. *N*-acylation dramatically influences the inhibitory activity of syringolin to proteasome [18]. As a promising anti-cancer agent, syringolin has great antitumor activity in inhibiting growth and inducing apoptosis of neuroblastoma, ovarian cancer cells, and other tumor cells [19, 20].

**Fig. 2:**
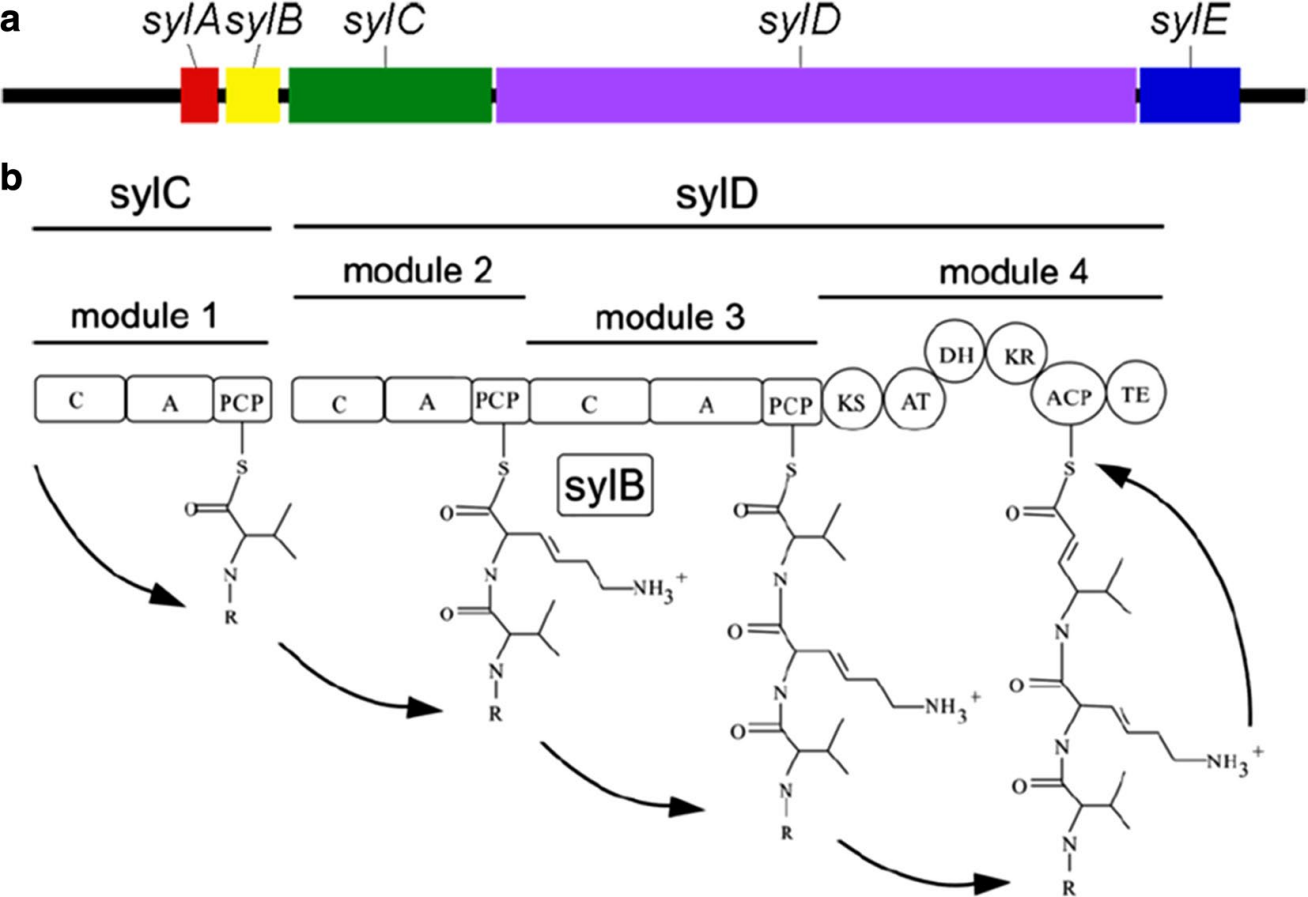
Arrangement of *syl* gene cluster and biosynthesis modules. Colored boxes represent open reading frames (ORFs) of *syl* gene cluster (**a**). Boxes labeled as C, A, and PCP represent condensation, activation, and peptide carrier protein domains of synthetase modules encoded by *sylC* and *sylD*, respectively. Circles denote putative β-ketoacyl synthetase (KS), acyl transferase (AT), dehydratase (DH), β-ketoreductase (KR), acyl carrier protein (ACP), and thioesterase (TE) domains of the polyketide-like module, which is also encoded by *sylD* (**b**)

The syringolin biosynthetic gene cluster spanned 22 kb in length and included five open reading frames (*sylA*–*sylE*). The NRPS module sequence in the chromosome generally determines the amino acid sequence of the peptide product [21] (Fig. 2) where *sylA* is a putative transcription activator; *syl*B hypothetically encodes the lysine reductase; *sylC* encodes a module predicted for valine activation [22]; *sylD* codes two typical NRPS modules that activate lysine (or dehydrolysine) and 5-methyl-4-amino-2-hexenoic acid (or its precursor); and *sylE* possibly serves as an exporter [23].

The genetic manipulation for PKS/NRPS gene cluster is difficult to perform using conventional DNA engineering methods because of their large sizes (spanning 10–100 kb). Red/ET recombineering [17, 18], which is independent of restriction site location and DNA fragment size, has extraordinarily advanced the field of genetic manipulation by omitting many steps in standard restriction/ligation. Direct cloning was recently established based on full-length Rac prophage protein, RecE, and its partner RecT-mediated linear plus linear homologous recombination (LLHR) [24]. This efficient cloning approach, when applied to direct cloning of large gene clusters from genomic DNA, might greatly promote the course of genome mining and combinatorial biosynthesis of PKS/NRPS compounds.

In this study, we report the direct cloning of an intact *syl* gene cluster from the genomic DNA of *Pss* B301D-R and replaced its native promoter with P_snpA_, a strong native promoter in *Streptomyces*, to actively produce the *syl* gene in heterologous hosts. We analyze the bioactivity of the recombinant products by treatment of several cancer cell lines and tumor model in mice to provide a suitable protocol for syringolin production and clinical application in the future.

## Methods

### Bacterial strains and culturing conditions

Various *E. coli* strains were cultured at 37 °C in Luria–Bertani (LB) medium supplemented with antibiotics. Kanamycin (30 μg/mL; Sigma Chemical Co., St. Louis, Mo.), blasticidin S (50 μg/mL), tetracycline (5 μg/mL), and ampicillin (100 μg/mL) were added to the growth media as required. Heterologous hosts, *S. lividans* TK24 and *S. coelicolor* M145 strains, were grown at 30 °C on M2 (0.4% glucose, 1% malt extract, 0.4% yeast extract, and 0.1% CaCO_3_) or in TSB medium (tryptic soy broth (Oxoid), 30 g/L) for metabolite analyses as previously reported [25]. MS-agar medium [14] was used to transfer the cosmids from *E. coli* into *Streptomyces* in accordance with the standard protocol [26]. Apramycin (50 μg/mL) and nalidixic acid (25 μg/mL) were supplemented in the medium whenever necessary.

### Recombineering

All engineering used Red/ET recombination techniques as described previously [27, 28]. Red/ET-competent *E. coli* cells (50 μL) were electroporated with 0.3 μg of a linear fragment (either PCR product or fragment obtained from restriction). PCRs were performed with Phusion polymerase (New England Biolabs, GmbH, Frankfurt am Main, Germany). After electroporation, the selection of recombinants was carried out depending on antibiotic-resistant gene and examined by restriction analysis.

### Direct cloning of the *syl* gene cluster

For direct cloning, genomic DNA from *Pss* B301D-R (GenBank: AJ548826) was digested with restriction enzymes, *Asc* I and *Sna*B I, to release the syl gene cluster region. *Nco* I and *Bam*H I digests of plasmid p15Adir [12], served as the PCR template. DNA Oligos used for generation of p15A-IR-Tpaes-BSD-oriT-IR backbone (Tpaes: MycoMar transposase gene; IR: inverted repeat; BSD: blasticidin S-resistant gene) were as follows: (sequence as homologous arm for recombineering is in lowercase)

Syldir5: 5′-ttgcactctgttcgaactcccattccagcttttgtctgccggtgcttttttcatggccaaccgtatcaccgcgcaatgaaagtg
  ccatcacATTTGATCCTCGTTATCTAG-3′;
Syldir3: 5′-tgcggcgaagctttgcatgacccagtgcagtacgtccgggtccagcagatgccattcgcgacgcgccttgaccaccgtg
  ccgacacgcggccAAGCTTGACCTGTGAAGTGAAAAATG-3′.

PCR product and linear genomic DNA were co-transformed into recombineering proficient competent GBdir cells to obtain p15A-syl-IR-Tpaes-BSD-oriT-IR. Sequencing the *syl* gene used primers sylseq-up (5′-ccggcctacacgcattc *sylA* end) and sylseq-down (5′-agcaacctggatgtacgg *sylE* end).

### Engineering the *syl* gene cluster

To obtain highly heterologous expression in *Streptomyces* strain, a strong promoter P_snpA_ was inserted in front of the *syl* gene in p15A-syl-IR-Tpaes-BSD-oriT-IR to form the p15A-syl-IR-Tpaes-BSD-oriT-IR construct. The P_snpA_-apra cassette (apra: apramycin-resistant gene) was prepared with Psnpsyl5 (5′-TTAATGATGTCTCGTTTAGATAAAAGTAAAGTGATTAACAGCGCATTAGCGCGCCTATCCTCCATGGTATAAATCG-3′) and Psnpsyl3 (5′-GGAATTAATCATCTGGCCATTCGATGGTGTCGGGTCATGTGAGCAAAAGGGAAGCCGCGGGAGTAATCCT-3′).

### Conjugation into streptomyces

The engineered *syl* gene cluster was introduced into the chromosome of *Streptomyces* strains by triparental mating using *E. coli* helper strain HB101 (pRK2013). The mating mixture was plated on MS agar medium and incubated at 30 °C for 18 h. These plates were overlaid with 1 mL of water containing 500 μg of nalidixic acid and 1 mg of apramycin and incubated further for 5 days at 30 °C. After two cycles of single-colony purification on selective plates, the ex-conjugants were tested by colony PCR (Taq-polymerase, Invitrogen) with the following primers below:

sylC-checkF: 5′-ATGAGCACGCACCAGCACGC-3′;
sylC-checkR: 5′-CATTCACCAACTGCCCTATC-3′;
sylD-checkF: 5′-GGAGCAGACTTACGGTCAGA-3′;
sylD-checkR: 5′-TAGCCAGCATATTTTCCAGC-3′;
sylE-checkF: 5′-TGGCGTTGACACTTTATTCA-3′;
sylE-checkR: 5′-CAACGTTACCCGCAAATATC-3′.

### RNA extraction and RT-PCR analysis

Total RNA was extracted by the TRIzol^®^(Invitrogen) method. The RNA quality was analyzed by absorbance measurement and formaldehyde-denatured agarose gel electrophoresis. RT-PCR of *syl* gene cluster was performed based on the previous protocol [29]. Control (RT-minus) reaction including all components for RT-PCR except the reverse transcriptase enzyme excluded the presence of genomic DNA. The expression of 16S rRNA gene from heterologous host served as a positive internal control. Reverse transcription reactions were conducted with the primers: sylB5 (5′-TGGCGCATGACCGATTGCGT-3′), sylB3 (5′-TCGGCATGCACGGGGACAAC-3′), sylC5 (5′-ACTGCCAATGGGAGCGCGAC-3′), sylC3 (5′-CAACTTACCCG GCAGCGGCA-3′), sylD5 (5′-ACTATCGCGCTCGTGTCCAA-3′), sylD3 (5′-CAGCCCGATACCGTCAGAAA-3′), sylE5 (5′-AAAGCCTTGCGGCCGAGCAT-3′), sylE3 (5′-AACCAGGAGCACGTCGCAGC-3′), 16SRNA-F (5′-CTACCTCAAGCAGATCGGCAAG-3′), and 16SRNA-R (5′-GATCAGGTC CAGGAACGCCATG-3′).

### HPLC analysis and mass spectrometry of syringolin

Recombinant *Streptomyces* strains were grown on an M2 medium for 7 days at 30 °C. For the metabolite analyses, supernatant cultures were extracted with equal volumes of ethyl acetate after centrifugation and dried in a rotary evaporator. The extracts were then dissolved in methanol and filtered (0.22 μm pore size). LC–MS/MS experiments were performed on LTQ XL hybrid mass spectrometer (Thermo Fisher Scientific, USA) coupled to a Finnigan LC system (Thermo Fisher Scientific). The extracts were subjected and desalted online in a reverse-phase pre-column (C18 Pepmap column, LC Packings) and resolved on a nanoscale C18 Pepmap TM capillary column (LC Packings) at a flow rate of 0.4 mL/min (solvent A = 0.1% formic acid in H_2_O; solvent B = acetonitrile and 0.1% formic acid; 0–15 min 95% A and 5% B to 95% B [linear gradient], followed by 5 min 5% A and 95% B). Detection was carried out in positive ion model, auto MSn. Syringolins were identified by comparing the retention times and MS2 data identified from the original producer.

### Bioactivity assays

Cell viability and death was determined by 3-(4,5-dimethyl-2-thiazolyl)-2,5-diphenyl-2H-tetrazolium bromide (MTT) assay for adherent B16, HeLa, 4T1, and MethA cells in 96-well plates as described [12, 25]. Cells were incubated with 10 or 20 μL syringolin extracts from *Streptomyces* strains for 48–72 h, and the optical density (OD) of each well was determined in an ELISA reader at 560 nm.

In vivo therapeutic assessment was carried out using 4T1 breast tumor model and B16 melanoma tumor model as described previously [1]. SPF female BALB/c and C57BL/6 mice, aged 6–8 weeks old, were purchased from the SLRC Laboratory Animal Company in Hunan, China. Animals were bred and maintained in SPF conditions and were kept for at least 3 days before use. Tumors in the fourth mammary pads of female BALB/c mice were established with 1 × 10^5^ 4T1 mouse breast tumor cells, and C57BL/6 mice were implanted with SC tumors by injecting with 1 × 10^5^ B16 cells on the mid-right side. After the tumor volume reached ~ 0.2 cm^3^, breast tumor model BALB/c mice were randomly assigned to seven groups, and C57BL/6 mice bearing B16 tumor were randomly assigned to four groups. Syringolin extracts were injected for every 2 days within a span of 10 days. Tumor weights were estimated using two-dimensional caliper measurements conducted thrice per week using the formula: tumor weight (mg) = (a × b^2^)/2, where a and b are the tumor length and width in mm, respectively. At a defined time, mice were sacrificed by cervical dislocation. However, moribund animals characterized by irregular respiration, tremors, absence of voluntary response to external stimuli, and coma were killed for humane reasons and considered as animals that died during survival experiments. All animal experiments were repeated thrice in this study. All animal experiments followed the National Institutes of Health Guide for the Care and Use of Laboratory Animals and obtained the approval from the Animal Ethics Committee of Hunan Normal University.

### Histology

Primary tumors, liver, kidney, and spleen from tumor-bearing and control mice were harvested and fixed in 10% buffered formalin. Standard hematoxylin and eosin (HE) staining procedures were employed for morphological assessment [30]. The paraffin embedded samples were cut into 5 μm sections, and every twentieth section was stained and examined by microscopy.

## Results and discussion

### Direct cloning of intact *sly* gene cluster

**Fig. 3:**
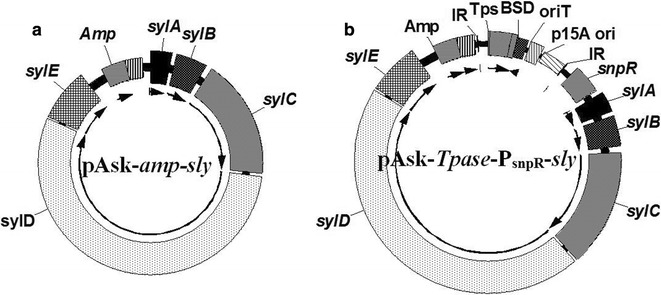
Plasmids constructed for *syl* gene heterologous expression. Direct Cloning vector pAsk-*amp*-*syl* (**a**) and the resulting plasmid p15A-*Tpase*-*syl* (**b**). Arrows represent the direction of transcription

The *syl* gene cluster from *Pss* B301D-R was isolated through Red/ET direct cloning. Plasmid pAsk-*amp*-syl (Fig. 3a) was constructed after PCR amplification of the minimal replicon with 90-nucleotide homology arms at the start and end of the *syl* gene. Digestion with *Pst*I and DNA sequencing revealed the presence of an intact *syl* gene. For successful integration into the chromosome and heterologous expression in *Streptomyces* strains, the resulting plasmid, p15A-*Tpase*-syl (Fig. 3b) was generated through triple recombination [31]. One fragment contained the *Bam*H I/*Xba* I restriction enzyme sites of p15Adir while the second one employed from PCR introduces a new promoter, P_snpA_, a regulator snpR, and another IR sequence. BSD- and kanamycin-resistant colonies were cultured and verified by restriction analysis. This direct cloning method led to less mutations and much longer target DNA fragments than the approach achieved by PCR, because their cloning depends on the *E. coli* replication machinery and not on PCR, which is error-prone. Obviously, the technique is easier and faster than the standard method needed to establish and to screen the genomic DNA library.

The recombinant *syl* gene cluster was introduced and was integrated into *Streptomyces* strains and chromosome through a well-established *E. coli: Streptomyces* intergeneric conjugation protocol [32]. Syringolin compounds are synthesized by enzymatic actions of the *sylB*, *sylC*, and *sylD* gene products, and the generation and condensation of the ureido valine remained enigmatic. The modified P_snpA_ promoter with native transcription-active *sylA* gene proved the efficient expression of the *syl* gene cluster in *Streptomyces* heterologous hosts.

### Identification gene transcriptional levels of *syl* gene cluster in heterologous expression hosts

**Fig. 4:**
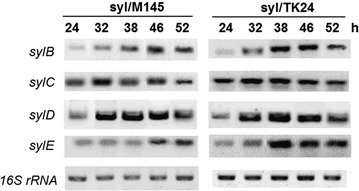
RT-PCR analysis for heterologous expression of *syl* gene cluster. RNA was isolated from the growth phase of syl/M145 and syl/TK24 cultures. Lanes 2–4 show the successful amplification of *syl* gene cluster from recombinant cultures. Lane 5 shows the amplification of the 16S rRNA internal reference

Transcriptional levels of recombinant *syl* gene in *S. coelicolor* M145 and *S. lividans* TK24 were evaluated via RT-PCR. Total RNA was extracted after fermentation for 24, 32, 38, 46, and 52 h. The *syl* gene cluster is well-expressed in both *Streptomyces* hosts (Fig. 4). *sylC* and *sylD* gene, which manages the extension of the core ring backbone, started the transcription process at 24 h. *sylB* catalyzed the reduction of the intermediate product. During the late fermentation period, *sylE* encoded thioesterase to release the syringolin compounds.

### Biosynthesis of syringolin in heterologous *Streptomyces* host

**Fig. 5:**
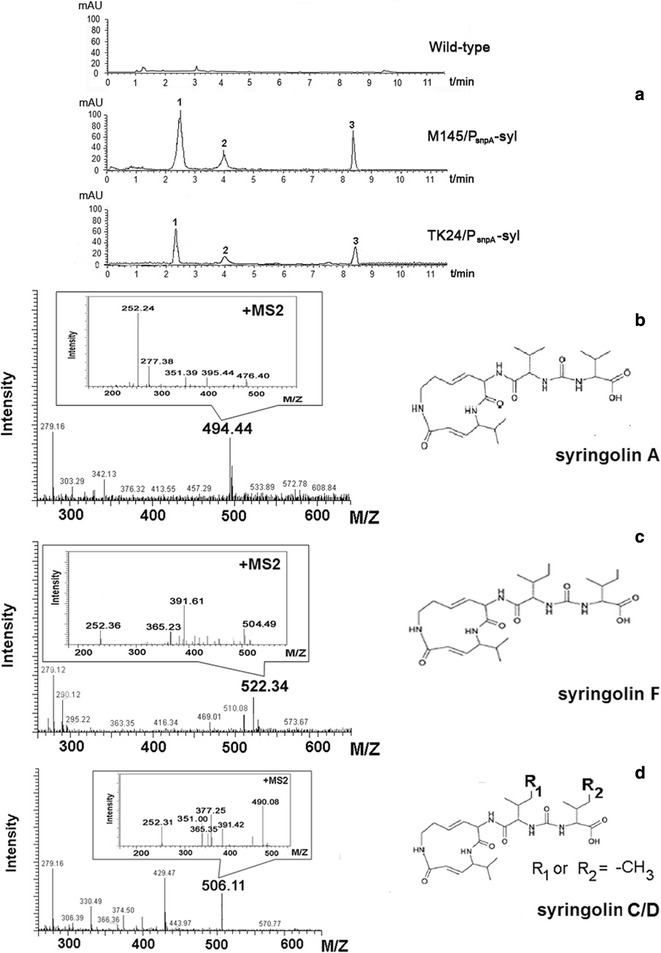
HPLC-MS analysis of recombinants. **a** Base peak chromatogram [BPC]; **b**–**d** MS and MS2 fragment pattern and chemical structure of syringolin compounds

HPLC analysis showed the presence of three metabolite peaks in the culture broth of both *S. coelicolor* M145/P_snpA_-syl and *S. lividans* TK24/P_snpA_-syl mutants in comparison with the individual native strains of *S. coelicolor* M145 and *S. lividans* (Fig. 5a). In order to compare the yield of syringolin compounds in *Streptomyces coelicolor* M145 and *Streptomyces lividans* TK24, we measured and calculated the peak area of the major derivatives produced by the software Bruker Compass. The results showed that the production of syringolin derived compounds produced by heterologous host *S. coelicolor* M145 was about 1.3-fold that of *S. lividans* TK24. This 1.3-fold difference in the syringolin yield of *S. lividans* TK24/P_snpA_-syl might have resulted from the diverse regulatory system in heterologous hosts. Overexpression of novG CstrR-like positive regulatory protein confers a higher PKS/NRPS synthesis activity to *S. coelicolor* M145 [33]. UV spectra, retention time (R_*t*_), and MS/MS2 results in HPLC/MS comparisons revealed that all known syringolins A, B, C, D, E, and F (SylA–SylF) were detected in the extract of the two heterologous production hosts (Fig. 5b–d). The three peaks represented C_24_H_39_N_5_O_6_ (syringolin A/B, **1**, R_*t*_ = 2.1 min, *m/z* 494 [*M* + H]^+^), C_26_H_43_N_5_O_6_ (syringolin F, **2**, R_*t*_ = 4.03 min, *m/z* 522 [*M* + H]^+^), and C_25_H_41_N_5_O_6_ (syringolin C/D/E, **3**, R_*t*_ = 8.37 min, *m/z* 508 [*M* + H]^+^). Low-resolution ESI–MS analyses further showed that the mass of **1** is lower by 14 and 28 amu compared with that of **3** and **2**, respectively. These results indicated that their real molecular weights of **1**, **3**, and **2** might be 493, 507, and 521 Da, respectively, suggesting the single methylene group (–CH_2_–) difference, preferably at the R_1_ or R_2_ group among the compounds. The molecular formula of the syringolin derivative obtained through high-resolution EI-MS matched the predicted structure.

### In vitro antitumor activity of recombinant syringolin compounds

**Fig. 6:**
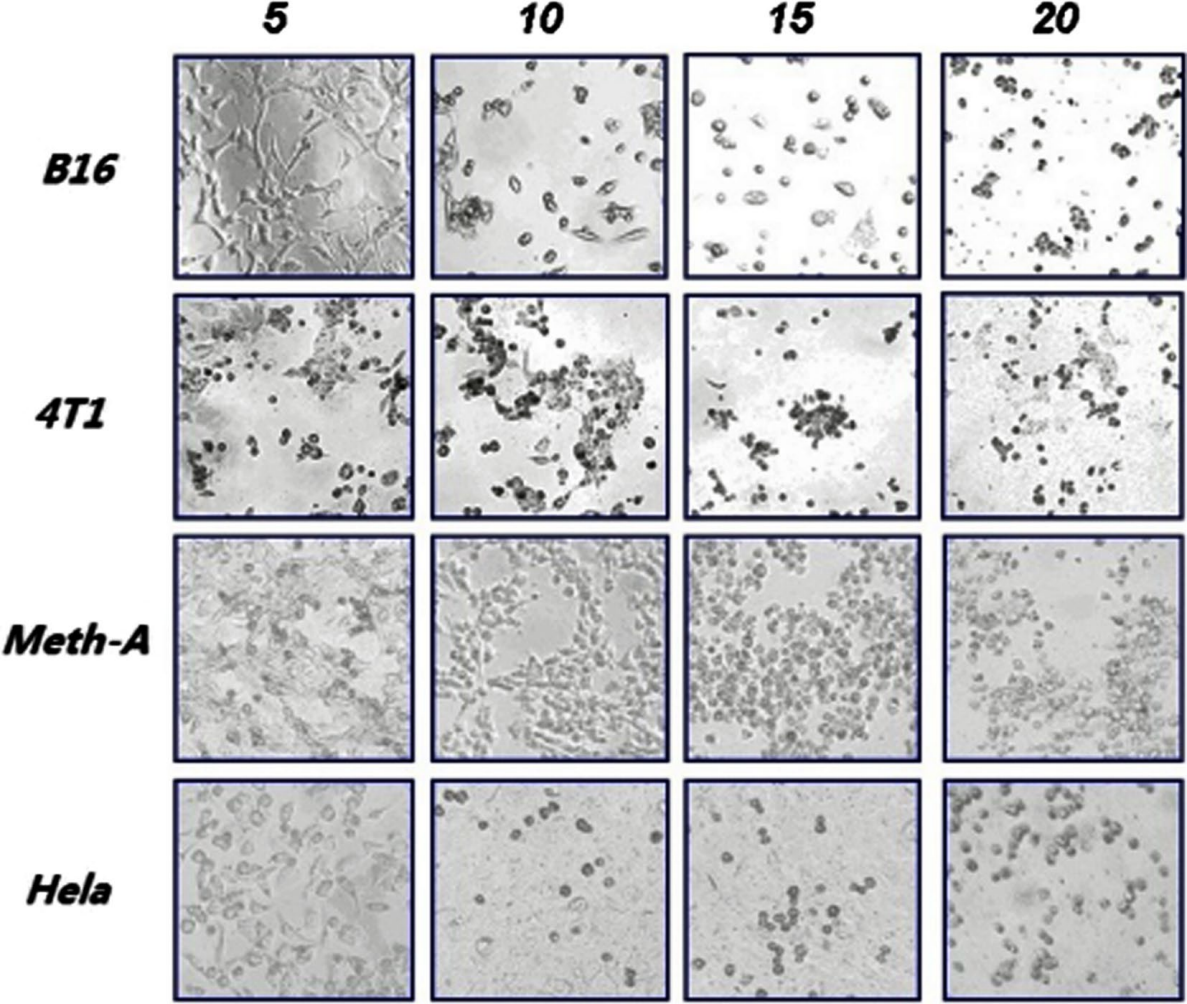
Inverted microscopy tumor cell. Nuclear morphological changes in syringolin-treated tumor cells after treatment with 5, 10, 15, or 20 μL of syringolin extracts

**Fig. 7:**
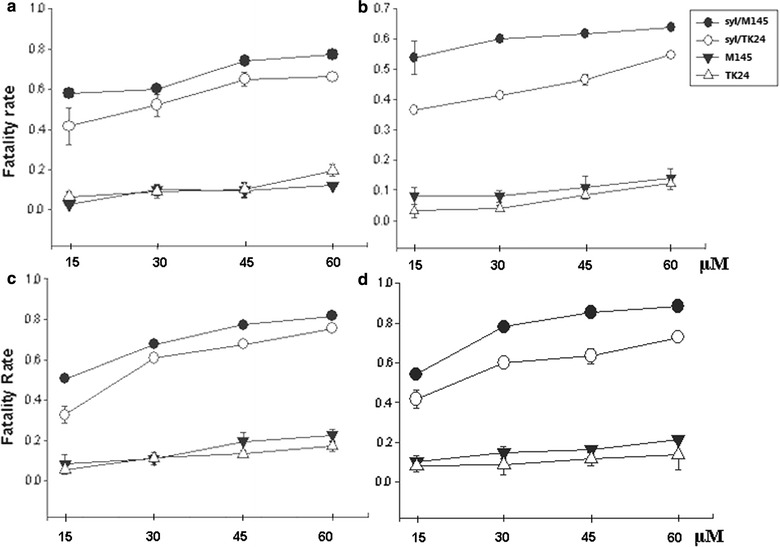
MTT analysis of recombinants. Fatality rate of HeLa (**a**), MethA (**b**), B16 (**c**), and 4T1 (**d**) after 24 h incubation with gradient syringolin extracts

We investigated the antitumor activity of recombinant syringolin on different tumor cell lines, and the results suggested that the extracts of the recombinant *Streptomyces* strains induced varying levels of cytotoxicity. All samples obtained from ethanol extraction methods were dried and resuspended in PBS, and the differences between the control and treatment groups were significantly and statistically different (*P* < 0.05), thus, confirming our results. Incubation of 4T1, B16, HeLa, and MthA cancer cells with 15, 30, 45, and 60 μM syringolin extracts for 24 h significantly reduced cell viability, as demonstrated by the reduction of MTT. Recombinant extracts showed dose-dependent cytotoxicity on all compounds. The effect of the extracts on cancer cells was evaluated (Fig. 6). Syringolin has IC_50_ values of 22.5, 24.3, 26.4, and 35.2 μM towards 4T1, HeLa, B16, and MthA, respectively. MTT analysis showed that the syringolin compound has good cytotoxicity to the above four cell lines at low concentrations (Fig. 7).

### In vivo antitumor activity of recombinant syringolin compounds

**Fig. 8:**
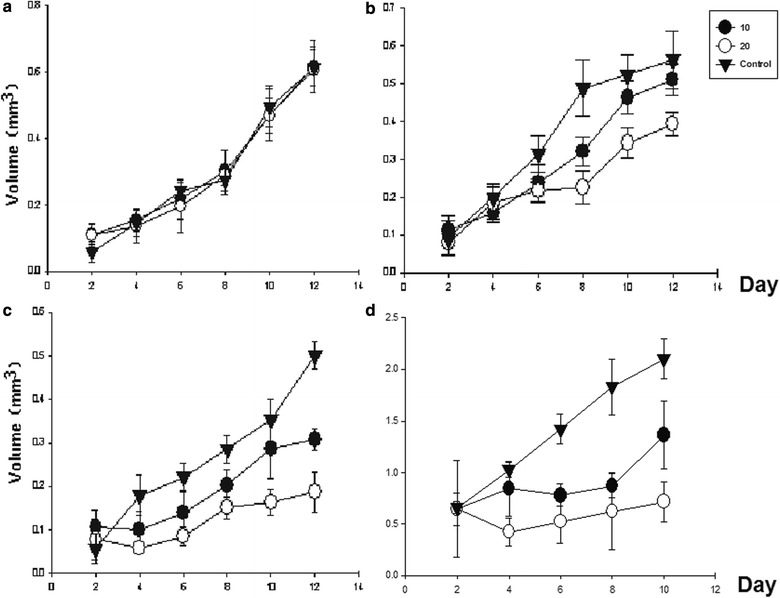
In vivo antitumor activity of syringolin extracts. Treated BALB/c mice bearing 4T1 breast cancer tumor by gastric lavage (**a**), intratumoral injection (**b**), and intravenous injection (**c**). Syringolin-treated B16 murine melanoma model in C57BL/6 mice by intratumoral injection (**d**)

**Fig. 9:**
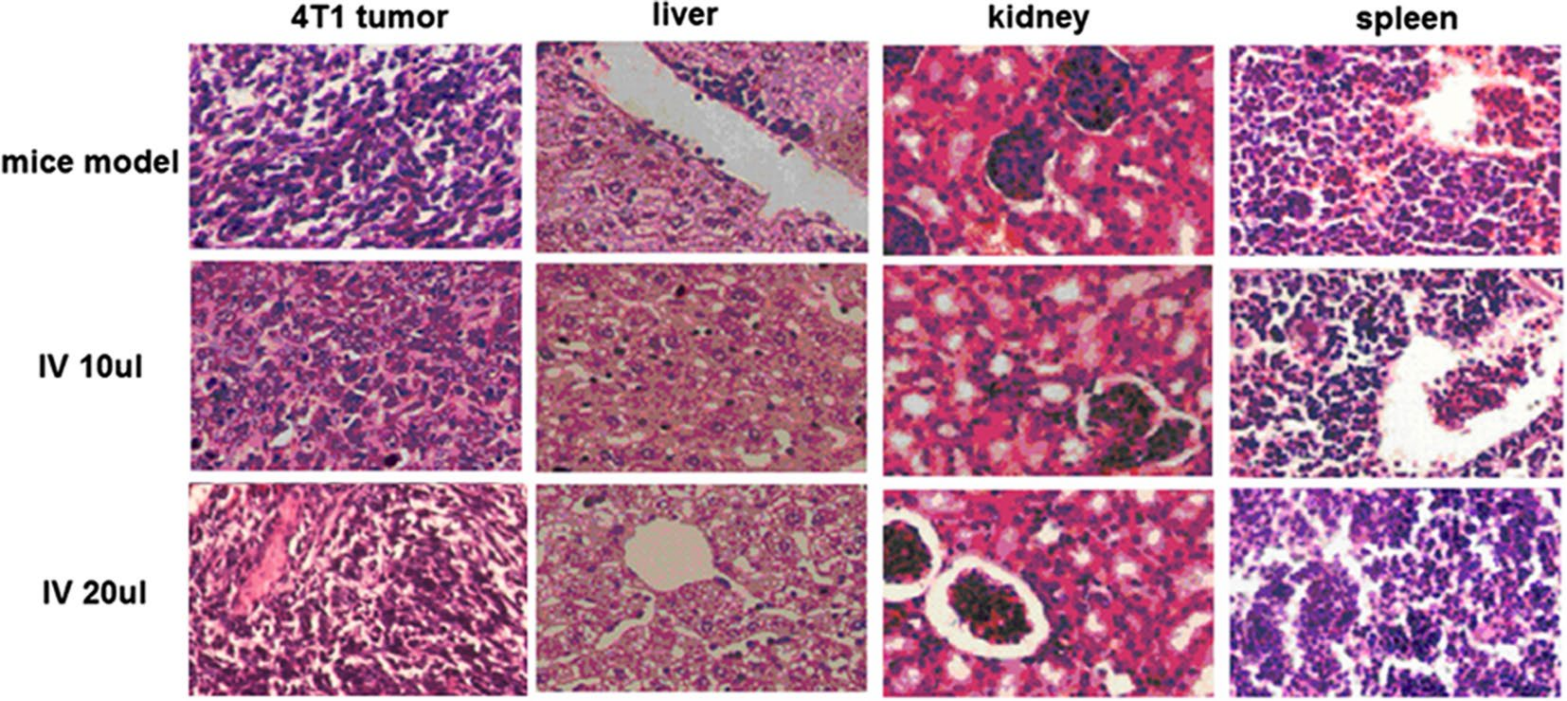
HE staining of section from main organs in mice bearing 4T1 breast cancer at ×200 magnification. Histological changes and apoptosis of BALB/c mice treated with 15 or 30 μM dose of syringolin extract by intravenous injection

We constructed two tumor models to analyze the in vivo toxicity of syringolin compounds. All mice were given 30 or 60 μM dose of syringolin extract every 2 days for 10 days. BALB/c mice bearing 4T1 tumor were treated with syringolin compounds through gastric lavage (Fig. 8a), intratumoral injection (Fig. 8b), or intravenous injection (Fig. 8c), and syringolin compounds had significant effect on 4T1 from the latter two methods (*P* < 0.05), but had no in vivo activity from former treatment. Among the three methods, intravenous injection gave the greatest effect with 67% in vivo inhibition rate to the 4T1 tumor at 30 μM, and extended mice mortality, resulting in delayed growth and death. Syringolin compounds by intratumoral injection could inhibit 40% tumor growth at higher concentration, and the injected mice survived under good conditions. Tumor, liver, kidney, and spleen were harvested and subjected to HE staining. Syringolin did not only increase apoptosis of 4T1 cells but also protected the liver and kidney from injury (Fig. 9). Organs from control mouse models showed significant liver and kidney damage while cells from the drug-treated group were normal and did not change much. Syringolin treatment of C57BL/6 mice bearing B16 tumor by intratumoral injection also showed great in vivo antitumor activity in a dose-dependent manner at 30 μM concentration with about 75% inhibition rate to B16 (Fig. 8d). B16 tumor growth almost stopped after injection of syringolin compound. Together, our results herein demonstrate the potential of syrinolin as effective anitumor agent that can treat various cancers without apparent adverse effects.

## Conclusions

This paper is first to express the whole *syl* gene cluster in heterologous *Streptomyces* strains. As *syl*A gene activates the expression of NRPS/PKS, acquisition of intact *syl* gene cluster uses the LLHR straightforward strategy mediated by Red/ET recombineering. The promoter underwent exchange after one round of LLHR. The results clearly indicate that the clusters of genes are capable of encoding proteins that synthesize syringolin. In comparison with the native promoter from *Pseudomonas syringae* pv. *syringae*, the general promoter, P_snpA_, successfully transcribed the whole gene cluster in heterologous strains. Large natural product biosynthetic gene clusters traditionally require reconstruction from several cosmids, which is time-consuming given the required screening process from a genomic library and subsequent cloning steps. Our direct-cloning method furnishes a general tool of reconstituting large gene clusters. When coupled with suitable heterologous expression hosts, direct cloning is effective alternative in investigating or engineering known and unknown biosynthetic pathways, from slow-growing bacteria and poorly established genetic systems.

Subsequently, the expression of the clone in both M145 and TK24 produce six syringolin family members, which show diverse transcriptions of the *syl* gene regulated by synthetic promoters. Syringolin yield is about 1.5 mg/mL. Replacement of some strong promoters, like *erm*Ep, *fdm*R1, or *nov*G might regulate the gene transcription. Another possible measure in developing the production is to optimize the fermentation of heterologous stains.

Syringolin derivants demonstrated high cytotoxicity to B16, 4T1, Meth-A, and HeLa cells in vitro and to 4T1 model BALB/c mice and B16 melanotic C57BL/6 mice in vivo. SylA could preferentially target the β2 and β5 of the proteasome in vitro and in vivo. Structure–activity analysis revealed that the dipeptide tail of SylA contributed to β2 specificity and identified a nonreactive SylA derivative being essential for imaging experiments. The syringoline family members showed their activities of labeling nuclear and cytoplasmic proteasomes in our research.

This research provided new avenues and ideas for the discovery and production of new antitumor compounds. The antitumor effects of syringolin may be attributable to the inhibition of proteinase and cancer cell invasion, and its concrete mechanism of inducing apoptosis in cancer cells still needs further studies.
